# Stretchable and reflective displays: materials, technologies and strategies

**DOI:** 10.1186/s40580-019-0190-5

**Published:** 2019-06-20

**Authors:** Do Yoon Kim, Mi-Ji Kim, Gimin Sung, Jeong-Yun Sun

**Affiliations:** 1 Department of Materials Science and Engineering, Seoul National University, Seoul, 151-742 South Korea; 2 Research Institute of Advanced Materials (RIAM), Seoul National University, Seoul, 151-744 South Korea

**Keywords:** Stretchable display, Reflective display, Active display, Passive display

## Abstract

Displays play a significant role in delivering information and providing visual data across all media platforms. Among displays, the prominence of reflective displays is increasing, in the form of E-paper, which has features distinct from emissive displays. These unique features include high visibility under daylight conditions, reduced eye strain and low power consumption, which make them highly effective for outdoor use. Furthermore, such characteristics enable reflective displays to achieve high synergy in combination with wearable devices, which are frequently used for outdoor activities. However, as wearable devices must stretch to conform to the dynamic surfaces of the human body, the issue of how to fabricate stretchable reflective displays should be tackled prior to merging them with wearable devices. In this paper, we discuss stretchable and reflective displays. In particular, we focus on reflective displays that can be divided into two types, passive and active, according to their responses to stretching. Passive displays, which consist of dyes or pigments, exhibit consistent colors under stretching, while active displays, which are based on mechanochromic materials, change their color under the same conditions. We will provide a comprehensive overview of the materials and technologies for each display type, and present strategies for stretchable and reflective displays.

## Introduction

**Fig. 1:**
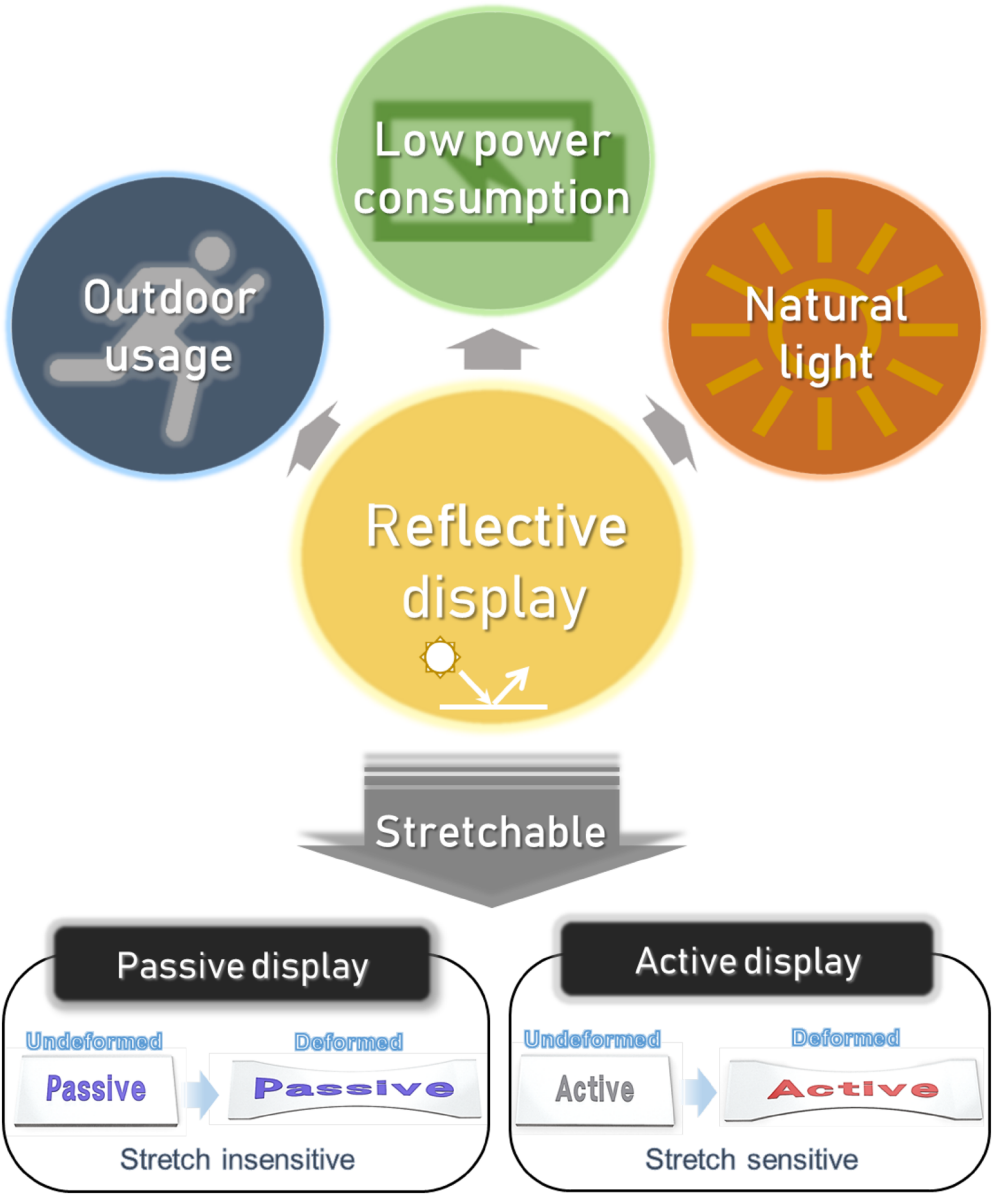
Schematic concept of reflective display. Reflective displays have advantages in outdoor usage due to their superior readability in bright condition. They consume less power than emissive display as energy for light generation won’t be needed. Reflective displays exhibit natural light with balanced wavelength spectrum, which is not enriched with short wavelength. Stretchable reflective displays can be classified into two groups, passive and active. Passive displays do not change their color when deformed, while active displays sensitively change their color due to deformations

Although emissive displays dominate the commercial mainstream, reflective displays have interested many researchers for a long time. Some customers are looking for reflective displays, namely E-paper, to read books more comfortably and ‘naturally’, even in the open air [1, 2]. Unlike emissive displays, such as light-emitting diodes (LEDs) and liquid crystal displays (LCDs), reflective displays do not contain internal light sources and use ambient light, mostly sunlight [3]. As they use the light from ambient space, they possess different properties to emissive displays (Fig. 1).

First, reflective displays require little consideration about health issues associated with blue light as they use natural light which is not enriched at short wavelength. Displays using LEDs are enriched at blue light region (400–490 nm) [4] Blue light is known to be harmful to health. It can provoke photoreceptor degeneration, age-related macular degeneration, and other types of eye damage under conditions of long-term exposure [4, 5]. LEDs are used for the majority of displays, such as TVs, phones and laptops. Furthermore, even LCDs, among other types of backlighted displays, use white LEDs due to their small size. Blue light is also suspected to contribute to sleep issues [6]. Reading light emitting E- books at evening showed more negative results to sleeping time and depth, compared to reading printed books at reflected light. It suppressed melatonin and changed circadian timing. Light emitting E-books commonly show much higher irradiance of blue light than printed books, and more blue light can increase alertness which disturbs deep sleep.

Second, reflective displays can achieve near-zero power consumption. In both emissive organic light-emitting diodes (OLEDs) and transmissive LCDs, power consumption is relatively high because they need to generate light [3]. Reflective displays do not need to generate light; they just reflect it; all that is needed is to control the wavelength of the reflected light.

Also, the readability of reflective displays is superior under ambient light [7]. The optical contrast of emissive displays tends to be severely diminished by bright ambient light. To deal with such problems, emissive displays need to increase their light intensity [8]. On the contrary, reflective displays’ readability is improved by bright ambient light; this makes them strong candidates for use as open air displays.

Outdoor use in everyday life is inevitable for most wearable devices. Reflective displays may be a good option for wearable devices due to their intrinsic advantages with respect to outdoor use. Even though Reflective displays are good options for wearable devices, existing reflective displays still need something more. They need to be stretchable. Stretchable displays are one of the key components for comfortable wearable devices [9], so reflective displays for wearable devices must attain stretchability.

Reflective materials can respond to stretching in two different ways. Some types of reflective materials exhibit a consistent color regardless of stretching, because they use specific dyes or pigments. Other materials change color when deformed, so they may possess different display characteristics compared to conventional displays. We can classify stretchable reflective displays into two groups, ‘passive’ and ‘active’, based on differences in their responses to deformation (Fig. 1). Passive displays maintain their color when stretched, like conventional displays, whereas active displays change color when stretched. In this review, we analyze features of both passive and active reflective displays, discuss their specific technologies and provide examples. Strategies for improving stretchability and addressing other issues associated with reflective displays will also be discussed.

## Passive reflective display

Passive displays maintain their color when they are deformed by bending or stretching, because they use dyes or pigments. For this group of reflective displays, electrochemical reaction or physical movement of material via an electric field is used. The intended color and image can be presented by controlling the electrical signal at each pixel. This is one of the most important properties of displays, ensuring that they show information clearly in every situation. Also, high resolution and speed can be expected due to their well-developed technologies. We now discuss some types of passive reflective displays that can potentially be made stretchable.

### Technologies for passive reflective display

#### Electrophoretic display

**Fig. 2:**
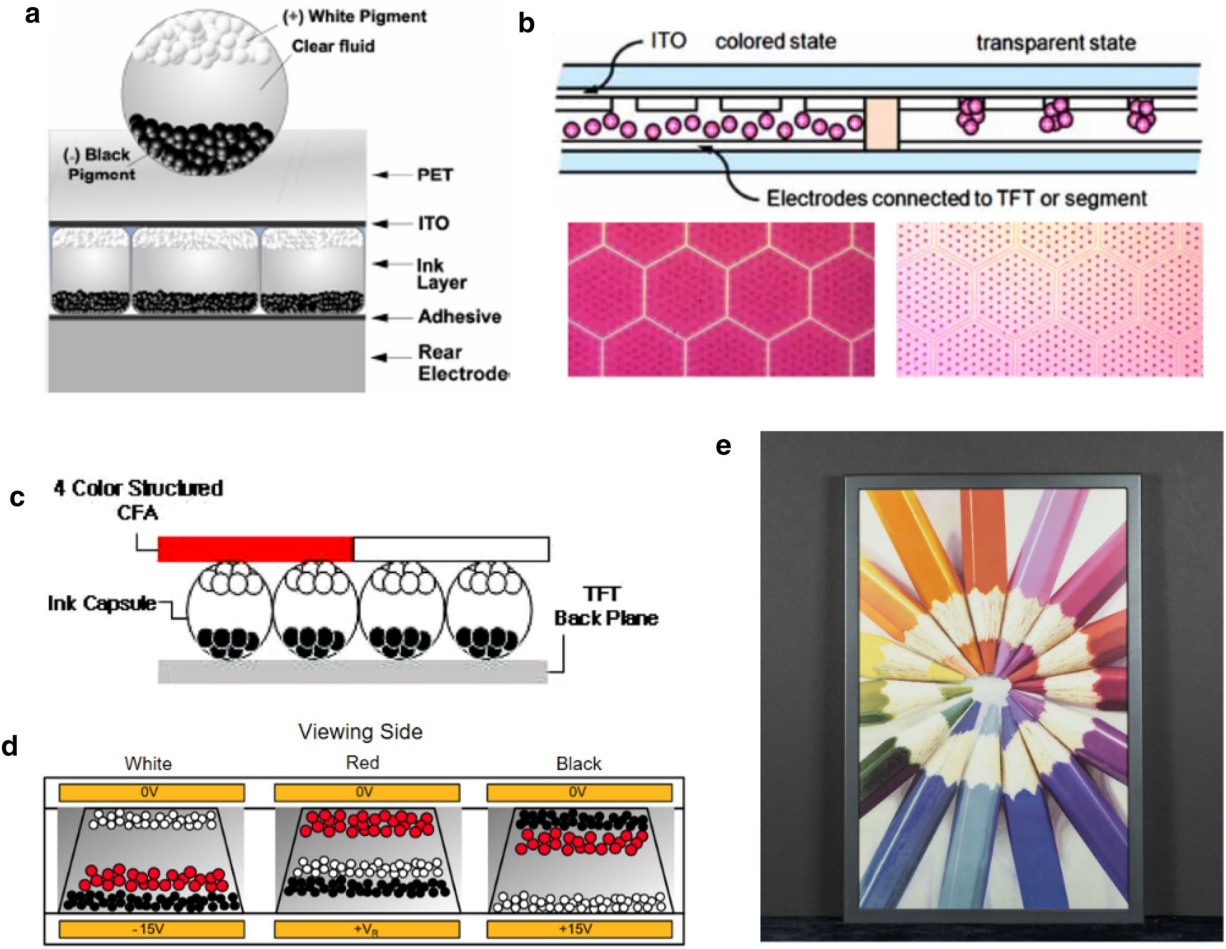
Technologies for electrophoretic display with their diagrams. **a** Cross sectional schematic of a microencapsulated electrophoretic imaging film and material of each part. **b** An electrophoretic display with hybridized vertical and horizontal movements of pigment particles. Microscopic images of display pixels are shown below. **c** An electrophoretic color display with color filter array. **d** An electrophoretic color display with three different pigment particles in one microcup. **e** An electrophoretic display produced by E Ink Holdings Inc. Pixel density of 150 ppi in 20 inch diagonal was demonstrated (Figure reproduced from **a** [107], Copyright 2012, The Society for Information Display; **b** [12, 13], Copyright 2012, The Society for Information Display and The Korean Information Display Society; **c** [20], Copyright 2008, Optical Society of America; **d** [21], Copyright 2018, IEEE; **e** [34], Copyright E Ink Holdings Inc.)

Electrophoretic displays control the color and brightness of each pixel by moving charged pigment particles. In conventional electrophoretic displays, particles migrate up and down as the direction of the electric field changes. Grayscale displays with two types of pigment, for example, have negatively charged white particles and positively charged black particles, which are suspended in clear dielectric fluid. If the white particles are on the side of the viewer, the pixel reflects white light so it appears white [10]. These particles are contained in microcapsules between a conductive, transparent top electrode and a series of rear electrodes (Fig. 2a). Indium tin oxide (ITO) glass is usually used as the top electrode because it is transparent and has good electronic conductivity. Titanium dioxide (TiO_2_) is mainly used to prepare particles due to its good chemical and optical properties [11].

Some grayscale e-paper devices, like the Amazon Kindle, have been launched successfully, but further effort is essential to broaden their applications. For example, the switching rate needs improvements for showing videos on electrophoretic displays. To improve the speed, a new mechanism using hybrid horizontal and vertical movement has been reported. Particles converge in dot-patterned cavities engraved in the plate (Fig. 2b). This device was demonstrated with a low working bias (< 15 V) and a relatively fast switching rate (< 300 ms) [12, 13]. Also, fast response times can be achieved by moving the pigment particles in gas. Electrophoretic displays which use charged powder can offer response time less than 0.2 ms but require relatively high voltages, from 40 to 70 V [14, 15].

There have been some reports on modifications of these particles. For example, chromaticity and density can be improved by coating ionic liquid polymer on the surface of porous silica nanoparticles [16–18]. Electrophoretic displays with these particles achieved response times of 155 ms at 0.2 mm thickness, which is faster than that of previously reported TiO_2_ and silica nanoparticles. Tridodecylamine has been studied as a charge control agent for various inorganic pigments and exhibits good optical properties with fast response times (220 ms at 200 kVm^−1^) [19].

Expression of various color is also an important factor in electrophoretic displays and it can be accomplished simply by covering grayscale displays with color filter arrays (Fig. 2c) [20]. However, this structure is not proper for vivid color because the area of pixel for each color(RGB or CMYK) is limited by filter. So, it can be improved by directly generating various color in on pixel without filter (key). So E Ink Holdings Inc. reported a three-particle electrophoretic display in one pixel to show specific colors in one pixel (Fig. 2d) [21]. Also, Advanced Color ePaper (ACePTM) with a four-particle system can display eight primary colors in each pixel, including yellow, magenta, cyan and white (Fig. 2e) [22].

#### Electrowetting and electrofluidic display

**Fig. 3:**
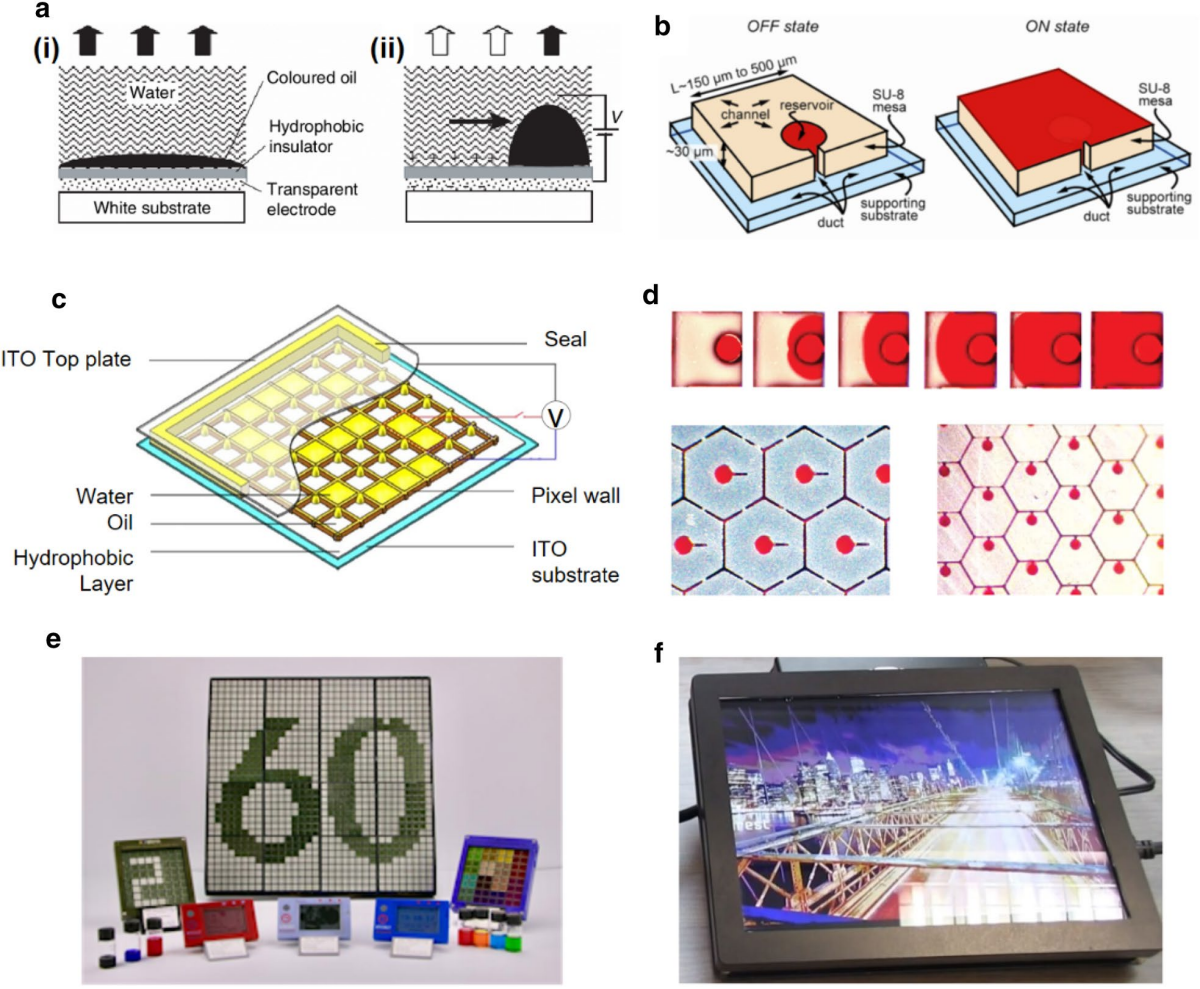
Technologies for electrowetting and electrofluidic display. **a** Structure and principle of electrowetting display. (i) Without any applied voltages, a colored oil film covers the pixel. (ii) When a voltage (~ 10 V) is applied, the oil film is contracting and makes the pixel transparent. **b** Schematic of an electrofluidic cell without a top plate. **c** Pixel array of electrowetting display with yellow dye based on alkylated pyrazole azo structure. **d** Images of electrofluidic pixels (square type and hexagonal type). Time-lapse images of a 500-um-square pixel is displayed. **e** Electrowetting based E-paper display by GR8 Optoelectronics Ltd. **f** Electrowetting based display demo by Liquavista (Figure reproduced from **a** [23], Copyright 2003, Springer Nature; **b** [25], Copyright 2012, The Society for Information Display; **c** [26], Copyright 2018, The Society for Information Display; **d** [24], Copyright 2009, Springer Nature; **e** Copyright 2017, Gr8; **f** Copyright Liquavista)

Electrowetting displays are based on the wetting effect of polar solvents under an electric field. One pixel consists of water, colored oil and an electrode coated with a hydrophobic insulator. With no voltage, the dyed oil covers the entire pixel area and shows its color (Fig. 3ai). When the voltage is turned on, oil forms a small droplet so the colored area decreases to expose the white background of device (Fig. 3aii) [23]. In electrowetting displays, ~ 20% of the area is always occupied by colored oil. Only the rest area can show the background color, as a result the difference of color between on/off state is limited [24, 25].

To overcome this challenge, a new approach for electrowetting displays has been reported, known as ‘electrofluidic’. Electrofluidic displays have reservoirs so that oil droplets converge to one point. The liquid pigment can be held in less than 5–10% of the visible area and the contrast ratio can reach ~ 20:1. The pigment is confined in the reservoir without voltage, but covers the entire pixel area when voltage is applied (Fig. 3b) [24, 25]. The 500-μm square and hexagonal pixels have been demonstrated. The reservoir comprises only 5% of the viewable area in the case of hexagonal pixels (Fig. 3d).

In electrowetting displays, various color expression can be achieved by utilizing variety of pigments or dyes. Appropriate dyes with high chroma and solubility are key to achieving good display performance. For example, yellow electrowetting dye with good solubility in non-polar solvent was achieved through the introduction of a long alkyl chain into pyrazole azo dye. A fast switching speed (17.8 ms), high aperture ratio (68.5 %), low threshold voltage (24 V), good light stability (240 h under accelerated conditions) and low backflow phenomenon have been reported (Fig. 3c) [26]. The wettability of the solvent on the surface also influences the performance of electrowetting displays. Several amorphous fluoropolymers, such as Teflon AF1600 and Hyflon AD60 have been studied as hydrophobic coatings for water/air and oil/water contacts [27]. Dealing with such factors appropriately, GR8 Optoelectronics Ltd. and Liquavista demonstrated electrowetting displays of various sizes for numerous applications (Fig. 3e, f).

#### Electrochromic display

**Fig. 4:**
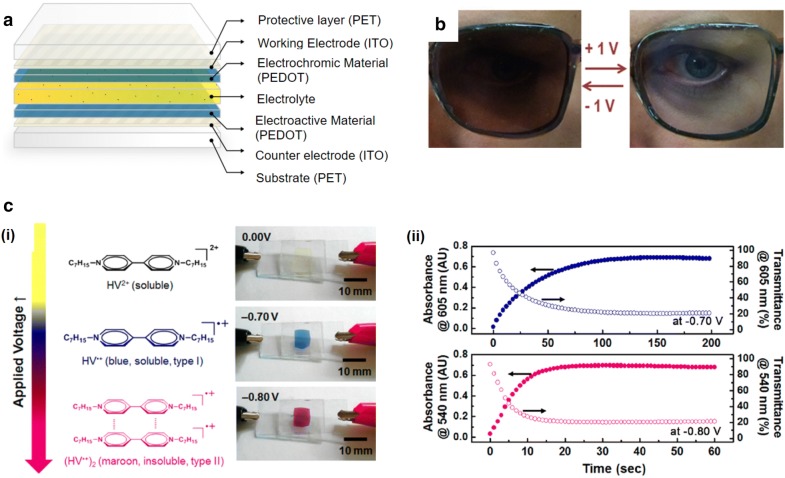
Technologies for electrochromic displays. **a** Assembly configuration of an electrochromic display and material for each part. **b** Photographs of an electrochromic glasses at − 1.0 V and + 1.0 V with brown blend of orange and periwinkle. **c** (i) Photographs for three different states of an electrochromic gel: bleached at 0.00 V, blue at − 0.70 V and red at − 0.80 V. (ii) Transient profiles in optical properties with constant applied voltages (Figure reproduced from **a** [28], Copyright 2015, The Author(s); **b** [30], Copyright 2015, American Chemical Society; **c** [31], Copyright 2016, American Chemical Society)

In electrochromism, the visible color changes due to electrochemical reactions. This functionality is possible due to the reversible redox reaction that changes the wavelength of the reflected light. Hence, electrochromic devices include electrochromic materials with electrolyte layers, and electrodes for redox reactions. These devices are typically assembled in a laminate configuration based on a simple two electrode configuration (Fig. 4a) [28].

There have been many attempts to apply electrochromic displays to commercial products, such as segmented polymer electrochromic displays [29] and user-controlled eyewear (Fig. 4b). Also, various colored active materials can be generated by mixing electrochromic molecules. For example, a brown blend of orange and periwinkle has been obtained and utilized for sunglasses; a transmittance change from + 1.0 and − 1.0 V was sufficient for this [30]. Multiple electrochromic states can be obtained in one device by controlling the chemical equilibrium. In the case of heptyl viologen (HV), cation radical monomers (HV^·+^) show blue color and dimers ((HV^·+^)_2_) appear maroon. The chemical state can be changed by applying different voltage (Fig. 4ci) [31]. However, coloration and bleaching time is a significant issue for various applications (Fig. 4cii). For high switching speeds and good durability, devices with viologen-anchored TiO_2_ nanoparticles and antimony-doped tin oxide (SnO_2_) nanoparticles were fabricated. These were stable even after 30,000 cycles driving at a speed of 4 Hz [32].

In short, the color variety and reaction speed of each pixel are important factors determining the performance of electrochromic displays. However, high switching speeds are only available in monochromic devices made of specific materials. To achieve applications beyond windows or glass, it is necessary to address these issues with electrochromic displays.

### Passive reflective display with flexibility

**Fig. 5:**
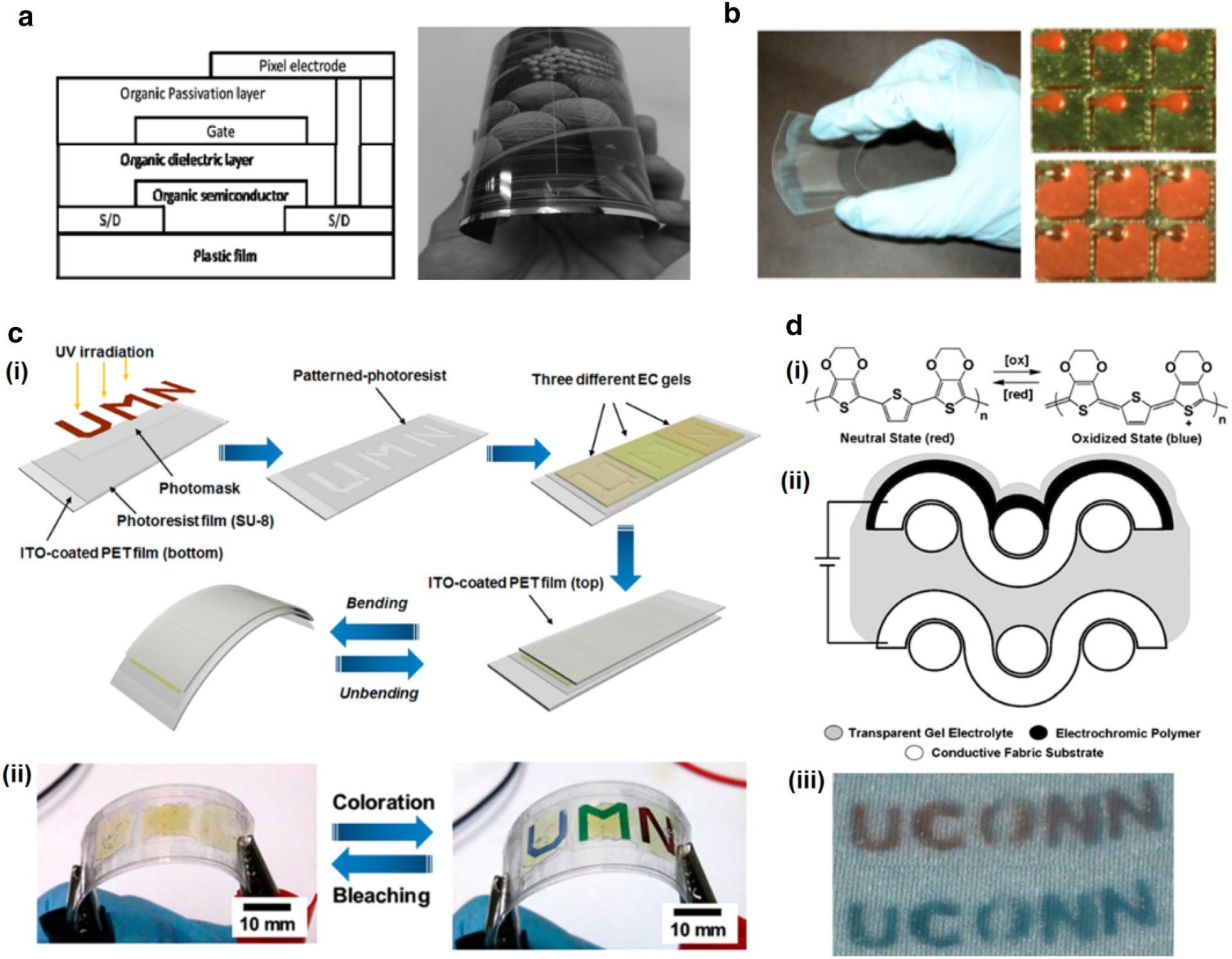
Passive reflective displays with flexibility. **a** Schematic diagram of the top-gate organic thin film transistors (OTFT) device structure. Flexibility of a 6′’ electrophoretic display is demonstrated. **b** A flexible electrofluidic display fabricated on a PET backplane and its pixels in the off and on states. **c** (i) Schematic illustration of fabrication processes for a flexible, patterned, multicolored electrochromic display on plastic sheet by using the ‘cut-and-stick’ method. (ii) Its bleached (at 0.00 V) and colored (at − 0.70 V) states under a bending deformation. **d** (i) PEDOT-PSS based electrochromic reaction. (ii) A schematic diagram of an electrochromic device on a fabric. (iii) An image of patterned electrochromic fabric in neutral (top) and oxidized (bottom) states (Figure reproduced from **a** [33], Copyright 2015, The Society for Information Display; **b** [25], Copyright 2010, The Society for Information Display; **c** [31], Copyright 2016, American Chemical Society; **d** [39], Copyright 2010, American Chemical Society)

Flexible electrophoretic technologies have been reported for the next generation of e-paper. One example is flexible electrophoretic display modules driven by organic thin-film transistor (OTFTs) backplanes on plastic film from E Ink Holdings (Fig. 5a). The optical performance of this 6′’ 166 ppi flexible module remained unchanged even after bending [33]. QR-LPD^®^ showed metal electrode PET base flexible display of 320 × 192, 80 ppi. Roll-to-roll manufacturing process applied to low cost PET films [18]. E-ink Mobius^TM^ is one of the best examples of a flexible electrophoretic display for commercial applications. Instead of fragile glass-based TFTs, it provides plastic-based TFT for flexibility, and can significantly reduce the incidence of display failure due to dropping or strain [34].

Also, electrofluidic pixels are particularly well suited to flexible display applications. Polymer backplanes, such as PET and poly (ethylene naphthalate) (PEN) backplanes, can be used as flexible base substrates. The structure is patterned by photolithography to create reservoirs and ducts (Fig. 5b) [25].

Electrochromic devices are among the most promising candidates for flexible passive displays. As ion gels have high ionic conductivity and good chemical stability at room temperature, they can contain electrochromic materials [35–38]. Multicolored flexible electrochromic devices were fabricated using ITO-coated PET film and demonstrated a color change at − 0.70 V with bending (Fig. 5c). This was accomplished by adding electrochromic viologen chromophores and a ferrocene electron source to the gels [31].

For another type of flexible electrochromic display, woven stainless mesh and Lycra spandex impregnated with poly (3, 4-ethylenedioxythiophene): poly (styrenesulfonate) (PEDOT-PSS) were used as conductive fabric electrodes (Fig. 5di, ii). Electrochromic polymers with gel electrolyte were prepared on the surface of the fabric electrodes. The electrochromic reaction in polymer displayed different colors between neutral and oxidized states [39] (Fig. 5diii).

## Active reflective display

Active displays change their color when stretched or compressed, and this feature is originated from mechanochromic materials that respond to mechanical stimuli. The mechanochromic materials visualize mechanical deformation with color changes and enable the active displays to be utilized as sensing agents. For examples, by being combined with human, the mechanochromic active displays can measure stress or strain exerted by human body and exhibit visual warning signs when they are highly stressed or strained. These characteristic make the display be readily exploited for the applications where real-time interactivity is more required than unilateral information delivery, such as sports industry or medical application. In this part, the mechanochromic reflective materials and driving mechanisms for active reflective display will be reviewed.

### Mechanochromic materials for active reflective display

#### Spiropyran embedded mechanochromic polymer

**Fig. 6:**
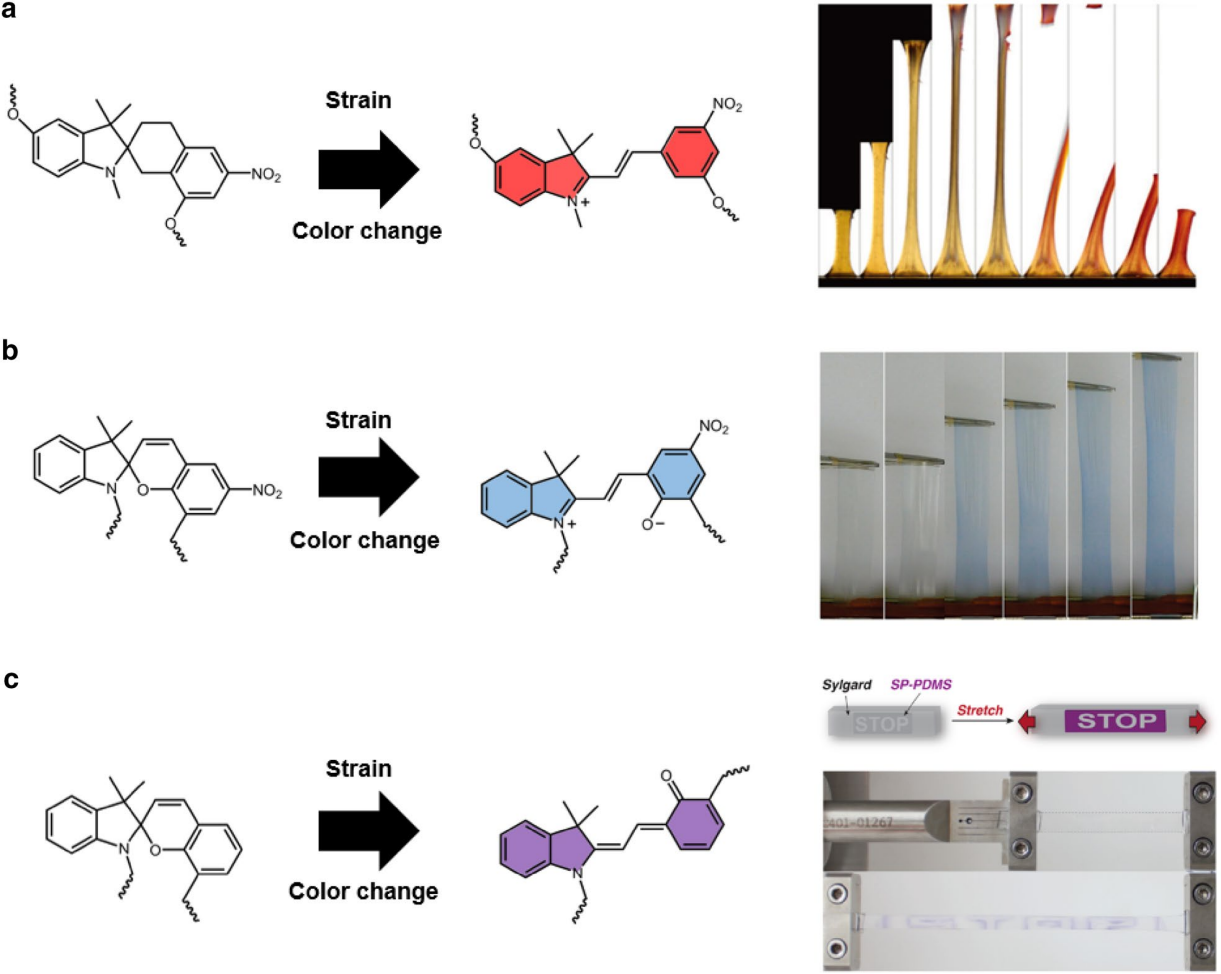
Mechanochromic spiropyran based polymers that change color after stretching. **a** Spiropyran(SP) embedded poly(methyl acrylate) exhibits color change from yellow to red by ring opening reaction induced by applied strain. **b** Indolinospiropyran-poly(e-caprolactone) films turned to blue colored merocyanine form by mechanical force-induced bonds rearrangements. **c** Spiropyran embedded PDMS elastomer is patterned on a PDMS substrate and show a word “STOP” after a deformation (Figure reproduced from **a** [50], Copyright 2009, Springer Nature; **b** [51], Copyright 2010, American Chemical Society; **c** [52], Copyright 2018)

Spiropyran is a well-characterized photochromic material that changes color upon UV exposure [40–43]. This material has been studied extensively, with features including responsiveness to various stimuli, i.e., temperature [44], pH [45, 46], solvent polarity [47], metal ions [48], and ultrasound [49]. In 2009, Davis and coworkers were the first to propose spiropyran-embedded mechanochromic polymers, in which mechanical force provides the activation energy for the chemical reactions necessary for color change [50]. The polymer was fabricated by directly linking spiropyran into the polymer chain of poly(methyl acrylate) or poly(methyl methacrylate) (PEGMA). By applying mechanical strain, the bulk polymer causes a ring-opening reaction of spiropyran with cleavage at the spiro C–O bond, which induces spiropyran to change into the highly colored merocyanine. Due to the change in molecular structure, a distinct red color gradually replaces the original yellow color as the polymer is stretched (Fig. 6a). Through a similar mechanochromic mechanism, a color change from colorless polymer to blue colored polymer was realized by O’Bryan et al. [51]. They embedded photochromic indolinospiropyran into poly e-caprolactone to obtain mechanochromic characteristics. The synthetic polymer undergoes transitions from spiropyran to blue-colored merocyanine due to mechanical force-induced activation (Fig. 6b). The sharp color transitions facilitate the use of bulk polymer for visible state detection, and the mapping of mechanical stresses upon strain. Moreover, to achieve highly noticeable visual signals, Barbee and coworkers exploited a patterning technique for mechanochromic polymers [52]. They fabricated a spiropyran-embedded polydimethylsiloxane (PDMS) elastomer that displays the word “STOP” in purple when a critical strain is reached (Fig. 6c).

#### 1-D photonic crystals as mechanochromic materials

**Fig. 7:**
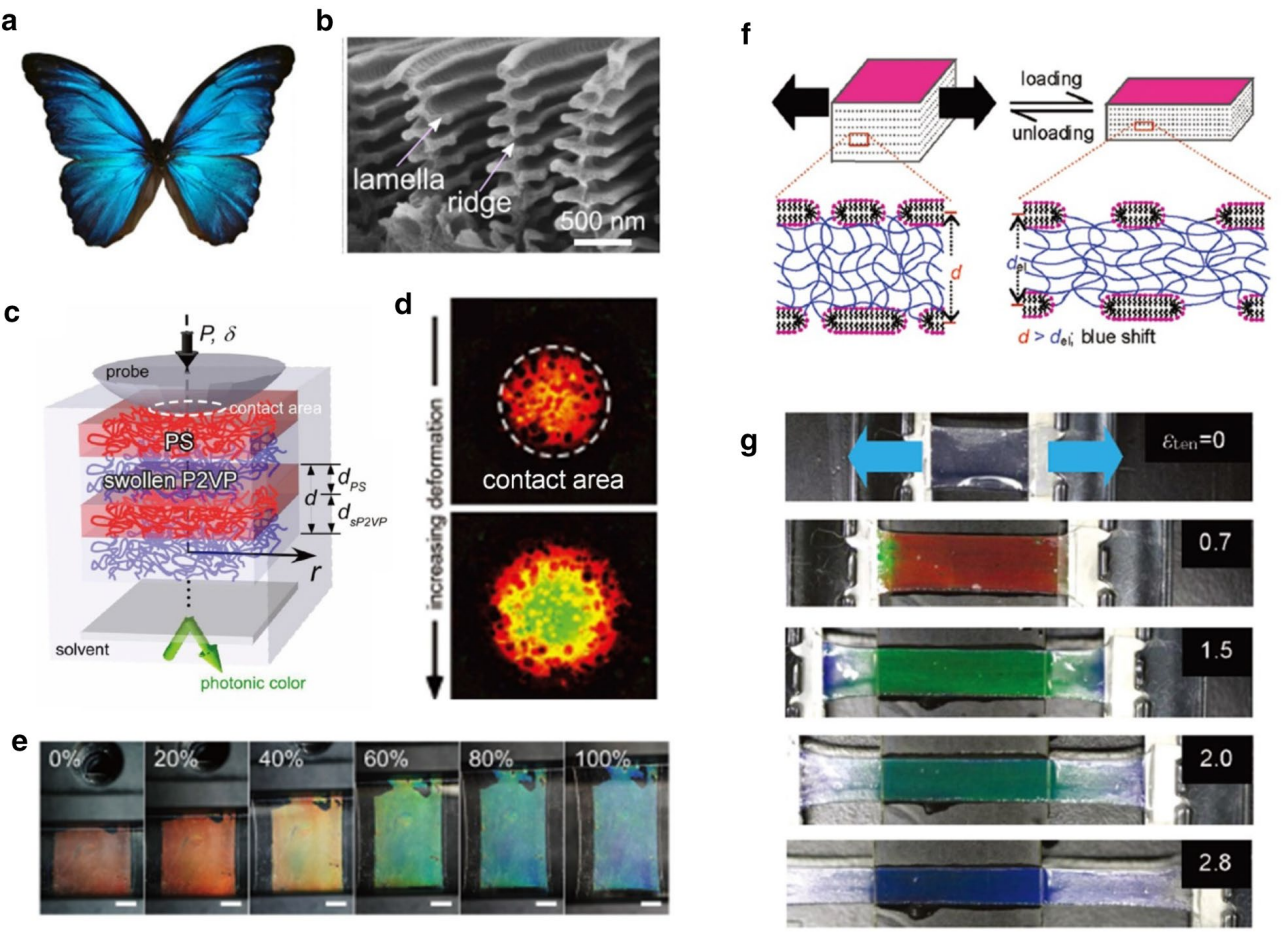
1D Photonic crystals for mechanochromic applications **a** Structural color from morpho butterfly wings. **b** Morpho ridges and lamella structures with alternating layers of air and natural material. **c** 1D photonic crystals with a PS-*b*-P2VP block copolymer. **d** Mechanochromic color changes of the block copolymer as a function of compression. **e** Block copolymer embedded PDMS elastomer changes color from red to blue by applied stretch. **f** Lamella structure of poly(dodecyl glycidyl itaconate) lipid bilayer/poly acrylamide hydrogel. **g** Mechanochromic demonstration of the hydrogel (Figure reproduced from **a** [53], Copyright 2003, Springer Nature; **b** [54], Copyright 2017, Springer Nature; **c**, **d** [59], Copyright 2011 WILEY-VCH Verlag GmbH & Co. KGaA; **e** [62], Copyright 2018, The Author(s), **f**,**g** [65], Copyright 2011, American Chemical Society)

Photonic crystals (PCs) have been considered attractive candidates because they can emit vivid and stimulating colors by modulating incident light at specific wavelengths (e.g., morpho butterfly wings and opals) [53]. It is well known that the colors of morpho butterflies are derived from wing ridges containing nanostructures, as shown in Fig. 7b [54]. There are alternating layers of air and natural material in the form of lamellas. Incident light is then reflected at the interfaces between the two materials, which have different reflective indices. Following constructive interference of specific wavelengths of light, a colored structure appears. To mimic the colors of nature, there has been intensive research on synthetic PCs in the form of one-dimensional (1D) and three-dimensional (3D) periodic structures. Due to the simple structures of 1D PCs, numerous studies have been reported on the use of techniques such as multilayer deposition [55–57] and focused-ion-beam-chemical vapor deposition (FIB-CVD) [58]. Self-assembled high-molecular-weight block copolymers (BCPs) have also been used as a material platform for creating 1D photonic crystal structures [59–62]. One of the most studied materials is polystyrene-b-poly(2-vinyl pyridine) (PS-b-P2VP). Kang et al. demonstrated a broad range of color-tunable lamella structures with alternating nonswellable glassy PS layers and soft swellable P2VP [60]. They exploited quaternization to induce conversion of the P2VP microdomains into swellable polyelectrolyte layers. These polyelectrolyte soft photonic layers contribute to mechanochromic behavior. Chan et al. demonstrated color changes in lamella BCP by applying mechanical stress that leads to changes in the soft P2VP layer due to compression (Fig. 7c, d) [59]. In the same manner, 1D BCP PCs that exhibit mechanochromic properties under stretching were demonstrated by Part et al. (Fig. 7e). They used PDMS elastomer as a substrate for the BCP layer and demonstrated that mechanochromic BCP achieves the full visible color range upon application of up to 100% uniaxial strain [62]. Another promising self-assembled system for 1D PCs is lamella structures of hydrogels (Fig. 7f). These photonic hydrogels contain a lipid layer made of hydrophobic poly(dodecyl glyceryl itaconate) (PDGI) bilayers in a polyacrylamide matrix (PAAm). By applying strain, the distance between the PAAm layer is easily tuned due to the soft properties of the gel. This leads to a dynamic color change from the transparent state to red, green and blue (Fig. 7g). Also, this hydrogel can support > 2000% strain due to the non-covalent hydrophobic associations of the lipid layer, which serve as sacrificial bonds for energy dissipation. This means that they can be readily utilized as mechanochromic materials [63–65].

#### 3-D photonic crystals as mechanochromic materials

**Fig. 8:**
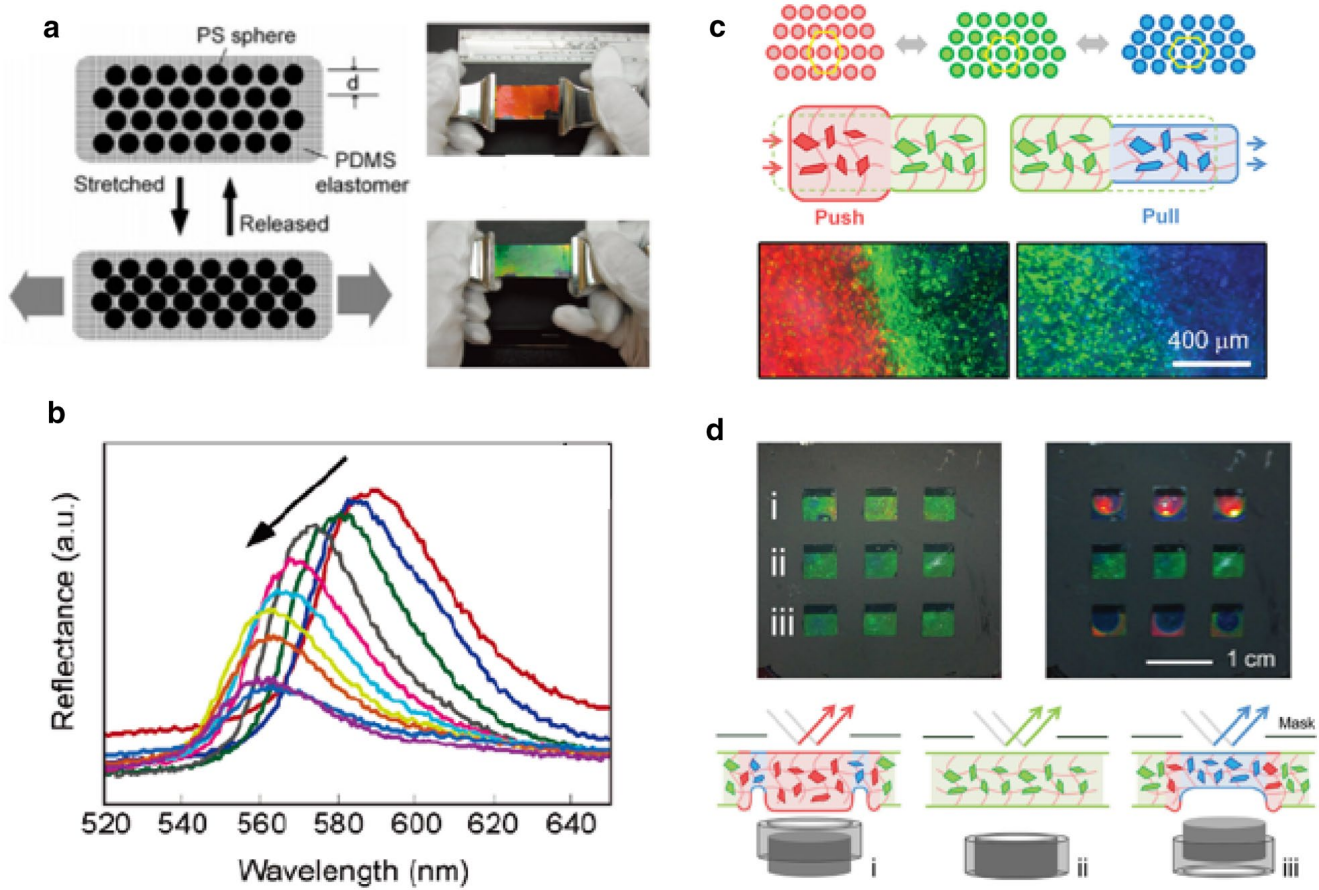
3D photonic crystals for mechanochromic applications. **a** Scheme of 3D photonic crystal with reversible lattice of polystyrene beads in a PDMS matrix. The lattice is controlled by stretching or releasing PDMS matrix. **b** Peak positions of reflected wavelength are shifted by elongating the photonic crystal embedded PDMS. **c** An illustration of silica particle arrays under deformation. Optical microscope images of 3D photonic crystal embedded PEGMA gel under pushing or pulling. **d** 3 × 3 pixels with the PEGMA photonic crystal gel (Figure reproduced from **a**, **b** [67], Copyright 2006, American Chemical Society; **c**, **d** [69], Copyright 2014, WILEY-VCH Verlag GmbH & Co. KGaA, Weinheim)

There have been many attempts to achieve mechanochromic behavior using 3D PCs in addition to 1D PCs [66–69]. Predominantly, 3D PCs are fabricated using synthetic opals with self-assembled polystyrene or silica colloids. The fabricated opal templates have subwavelength periodic structures that exhibit the reflected interference color, but the color from the opal structure does not change reversibly due to plastic deformation. To achieve color tunability, Fudouzi et al. [67] infiltrated elastomeric precursors into opal templates and then crosslinked them to ensure reversible color change upon mechanical stimulation. The polystyrene beads were embedded in PDMS, which was swollen to keep the particles separate. This ensures that the PC elastomer can modulate the interparticle distance easily and reversibly upon mechanochromic color change (Fig. 8a, b). Yang et al. [69] tuned the lattice distance sensitively by using soft gel materials as matrices for opal structures. They prepared highly responsive mechanochromic photonic gels by embedding SiO_2_ particles in poly (ethylene glycol) dimethacrylate (PEGDMA) gel and demonstrated its mechanical sensitivity and reversibility (Fig. 8c). By applying various mechanical stimuli, such as pushing, pressing and bending, photonic gels sequentially exhibit red, green and blue colors due to their mechanochromic properties (Fig. 8d).

### Electroactive reflective displays using mechanochromic materials

#### Electrochemical swelling driven color change displays

**Fig. 9:**
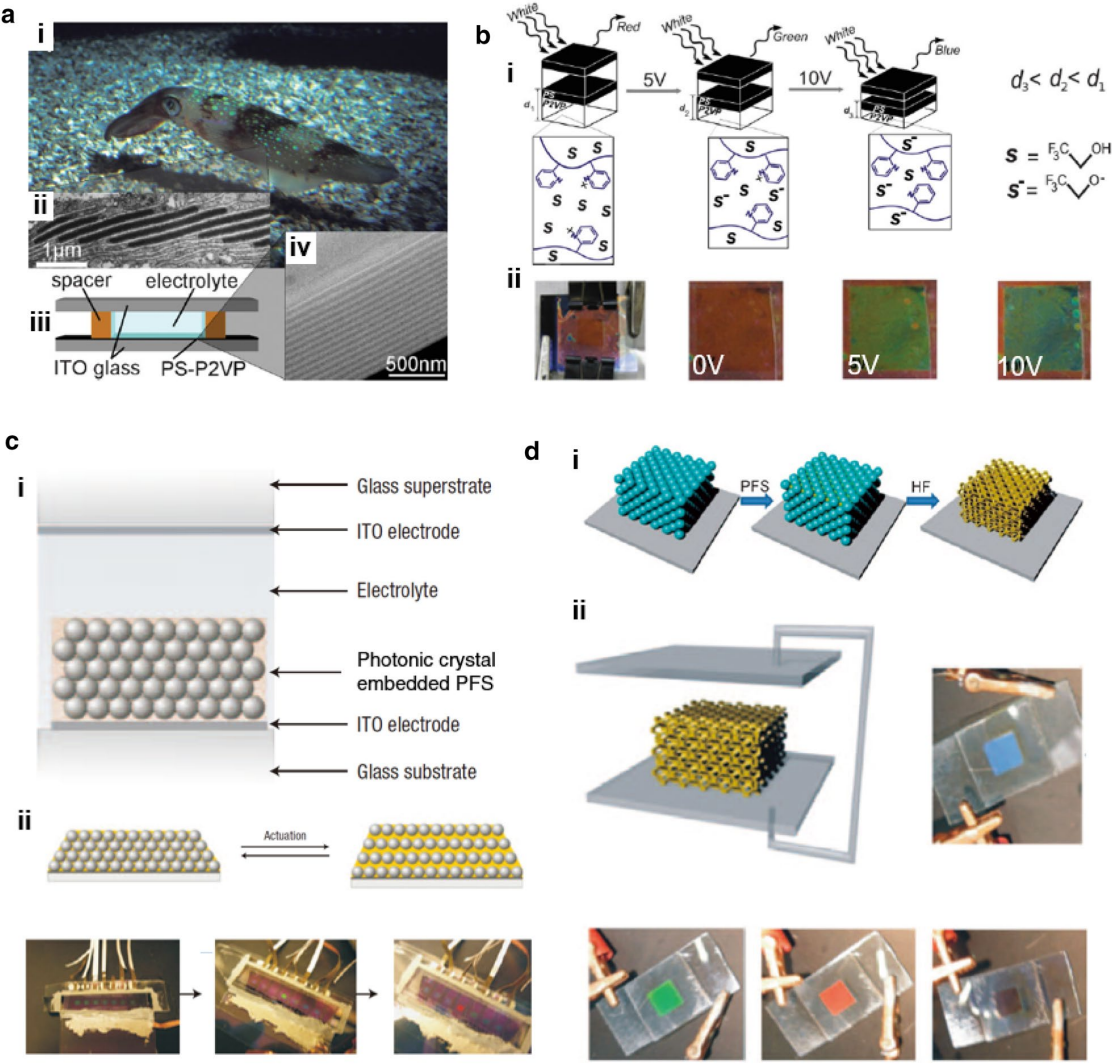
Electrochemical swelling driven color changes in photonic crystal systems. **a** Bio-inspired electroactive color changing 1D photonic crystal display. (i) Digital image of a cephalopod, (ii) protein based multilayer, (iii) A simple electrochemical cell is fabricated by introducing an electrolyte and lamella photonic gel between two ITO coated glass substrates. (iv) a lamella structure of PS-b-P2VP block copolymer. **b** Mechanism of the electrochemical color change, and optical images of the device with various electric potentials. **c** (i) Schematic of an electrochemical cell with 3D opal photonic crystals in polyferrocenylsilane (PFS) matrix. (ii) Electrochemical induced swelling leads to color change in the photonic crystal. **d** (i) Preparation process of PFS-based inverse opal structure. (ii) The inverse opal based photonic crystal is operated electrochemically (Figure reproduced from **a**, **b** [70], Copyright 2009, WILEY-VCH Verlag GmbH & Co. KGaA, Weinheim; **c** [73], Copyright 2007, Springer Nature; **d** [74], Copyright 2009, WILEY-VCH Verlag GmbH & Co. KGaA, Weinheim)

There have been many approaches to the integration of PCs into devices, where electrical signals can be used to control reflective colors easily and finely, in a continuous manner, for technological applications. One such approach is to exploit electrochemical reactions, which induce swelling of the soft layers of PCs, thus resulting in color change [70–74]. Walish et al. presented a bio-inspired electroactive PC display using electrochemical reactions [70]. They utilized a PS-b-P2VP BCP as a 1D PC to imitate cephalopod multilayer proteins that can change color dynamically. The color tuning on a synthetic PC progresses in a simple electrochemical cell containing lamella gel that is swollen in an electrolyte and sandwiched by an ITO-coated glass substrate (Fig. 9a). When voltages are applied to the cell, the trifluoroethanol (TFE) electrolyte is converted into trifluoroethoxide ions (TFX^−^) at the interface of the electrode, which decreases the thickness of the P2VP layer because TFX^−^ does not swell the P2VP domains (Fig. 9bi). As the voltage increases, the initial red color gradually changes into the shorter wavelength colors of green and blue due to shrinkage of the P2VP layer (Fig. 9bii). Likewise, electrochemical swelling can be applied to 3D PCs to change the color displayed. An electrochemical cell based on a silica opal array in polyferrocenylsilane (PFS) was presented by Arsenault et al. (Fig. 9ci) [73]. They used electrochemical reactions to oxidize PC-embedded PFS matrix, resulting in the loss of electrons. Subsequently, to neutralize the positively charged components of the PFS, anions from the electrolyte are driven into the oxidized PFS matrix. Due to the influx of ions, the PFS matrix becomes more swollen with the solvent, which leads to a change in the inter-particle distance, thus causing a color change (Fig. 9cii). To improve the color switching time of the 3D photonic electrochemical system, Daniel at all exploited a 3D inverse opal structure [74]. Compared to the opal structure, the electrolytes can easily permeate the inverse opal due to the absence of particles. The preparation process for the inverse opal polymer was based on etching of the silica particles in PFS matrix by hydrofluoric acid (Fig. 9di). Then, the inverse opal layer was introduced between the two electrodes, including an electrolyte. When voltages were applied to the integrated cell, the electrochemically driven swelling caused various colors to be displayed (Fig. 9dii).

#### Electrokinetic driven reflective displays

**Fig. 10:**
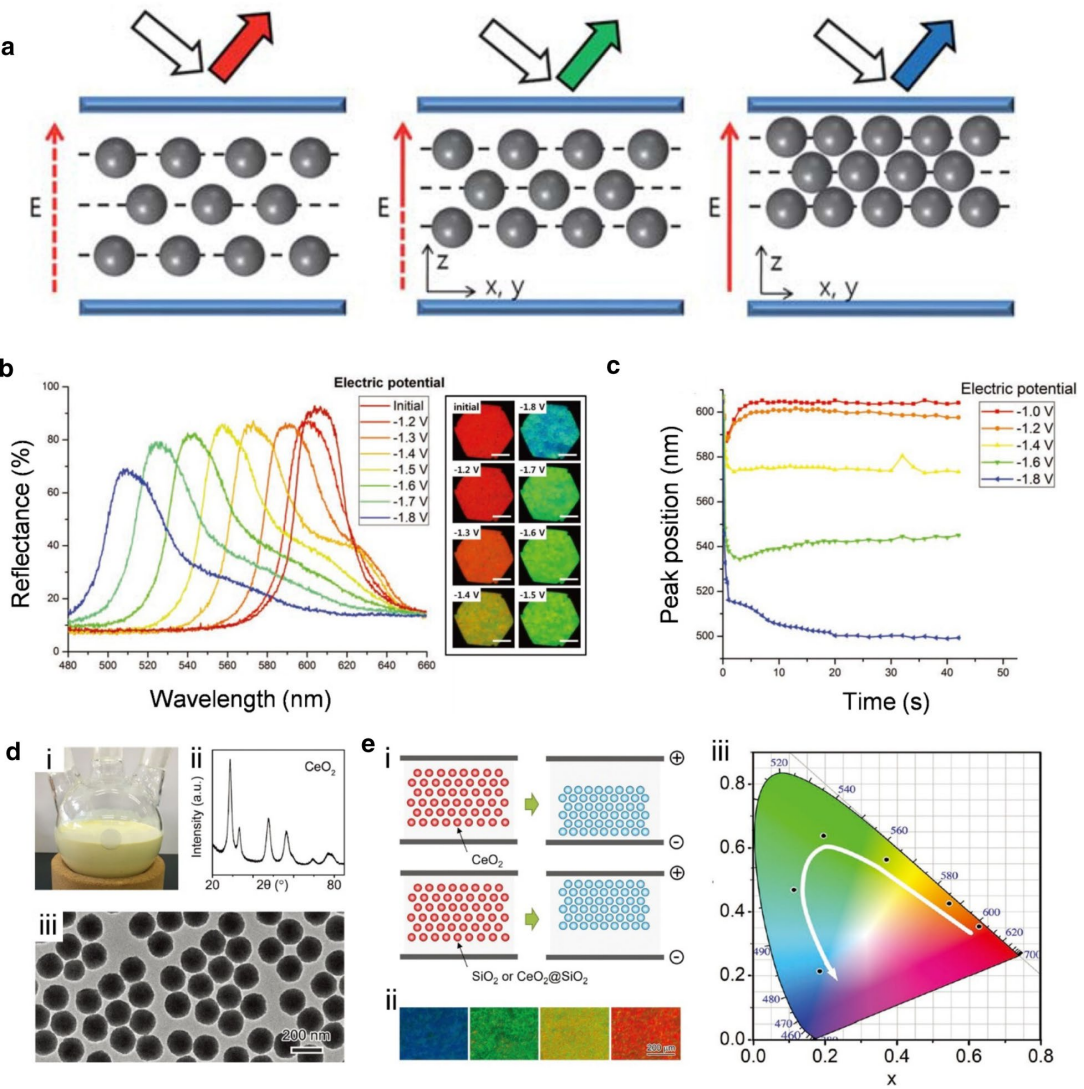
Electrokinetic driven 3D photonic crystals **a** Schematic representation of the changes in an array of charged particles under different electric fields. **b** Changes in reflective colors exhibited by a single device with various voltage bias. **c** Profiles of peak reflectance as a function of time under different DC bias excitation. **d** (i) A digital photo of CeO_2_ particles. (ii) a X-ray diffraction pattern of CeO_2_ particles. (iii) a TEM image of CeO_2_ particles. **e** (i) Working mechanism of the electrically tunable photonic crystals prepared from propylene carbonate solution of CeO_2_, SiO_2_, and SiO_2_ coated CeO_2_ colloidal particles. (ii) Optical microscope images for the photonic crystal under various voltages. (iii) CIE color space showing tunable range of photonic colors in different electric fields (Figure reproduced from **a** [76], Copyright 2013, The Royal Society of Chemistry; **b**, **c** [75], Copyright 2010, WILEY-VCH Verlag GmbH & Co. KGaA, Weinheim; **d**, **e** [77], Copyright 2018, WILEY-VCH Verlag GmbH & Co. KGaA, Weinheim)

Electrochemical swelling-driven systems tend to have long response times because they are governed by solvent diffusion inside polymers. The diffusion processes can be very slow, even in a bulk sample. Distinct from these systems, electrokinetic-driven systems have been developed with relatively faster color change responses [75–78]. Electrokinetic photonic systems have highly charged photonic particles in a liquid medium and the interparticle distance is controlled by an electric field (Fig. 10a). When the electric field is applied, charged particles are attracted to an electrode until the repulsion force between the particles compensates for the electrokinetic force induced by the voltage, which causes the color to change. These electrokinetic-driven color changes were demonstrated with sulfonated PS beads by Shim et al. Reflective colors are delicately tuned by controlling the applied direct current (DC) voltages (Fig. 10b). The peak positions of the reflected wavelength were measured as a function of time under different DC bias excitations (Fig. 10c). When the voltages were applied to the device, the reflection peak was shifted almost instantly, i.e., within 1 s, and the color became saturated after several seconds [75]. In addition to the faster responses and wider photonic colors of electrokinetic-driven systems, there have been attempts to exploit particles with high dielectric constants. It is well known that the high dielectric constant of a particle (except a magnetic particle) contributes to the high migration speed of charged particles and enhances the light scattering at the interface between particles and a medium due to a large refractive contrast. Fu et al. demonstrated faster electrical responsiveness and a broader spectrum of colors by exploiting cerium oxide (CeO_2_) particles in an electrokinetic photonic system. A monodisperse CeO_2_ particle was synthesized and analyzed by X-ray diffraction (XRD) and transmission electron microscopy (TEM) (Fig. 10d). Through XRD and TEM, we ensured that CeO_2_ particles had a face-centered cubic crystalline structure with a high dielectric constant and an average particle diameter of 154 nm. As the particle surface of CeO_2_ is positively charged during the synthesis process, CeO_2_ particles in propylene-carbonate liquid medium move towards the cathode when an electric field is applied (Fig. 10ei). For precise control of particles in electrokinetic systems, CeO_2_ was coated with a negatively charged silica thin layer. The silica-coated CeO_2_ particles exhibit highly saturated structure colors with broad color tunability, as shown in CIE color space, when different voltages are applied (Fig. 10eii, iii) [77].

#### Electromechanically driven color change displays

**Fig. 11:**
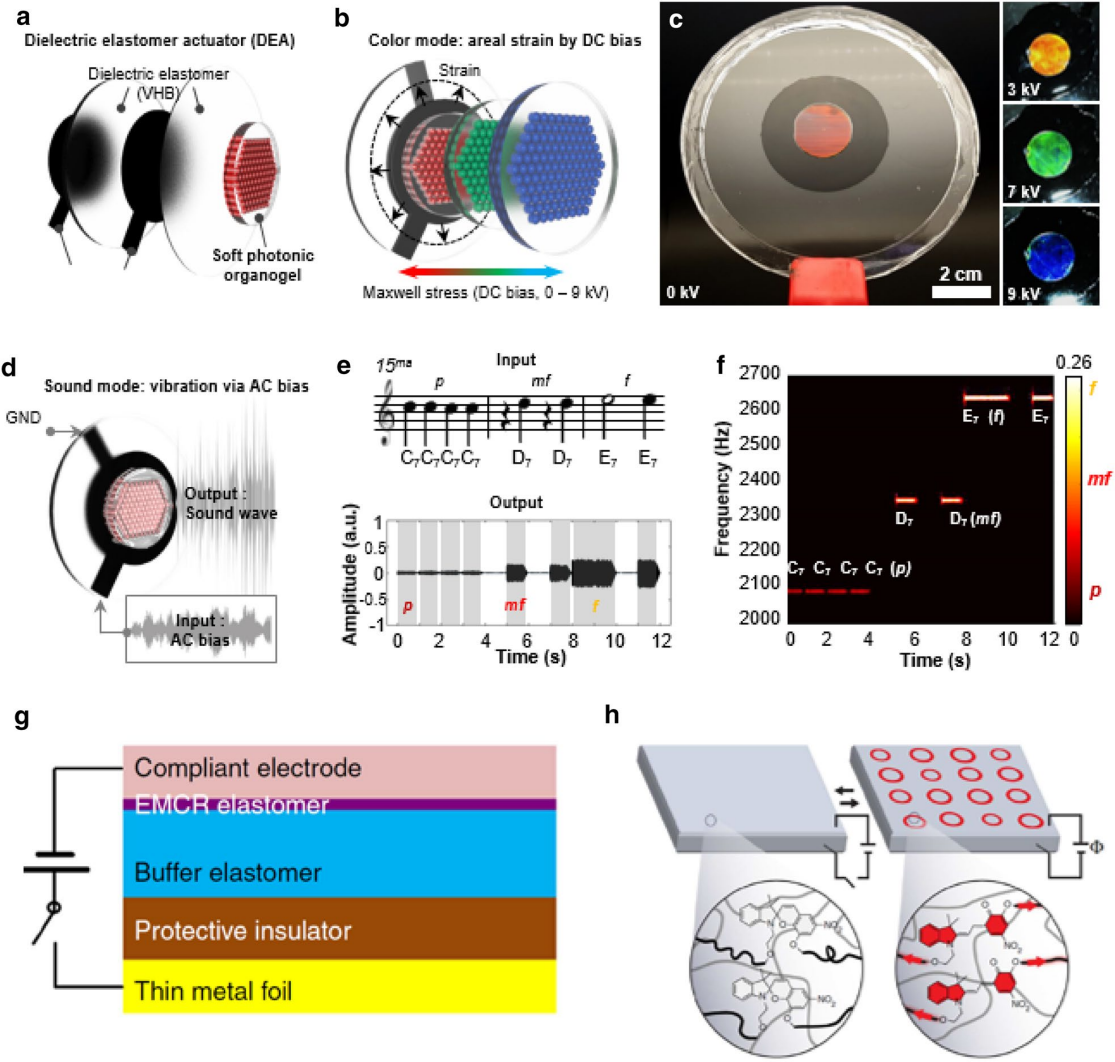
Electromechanically driven mechanochromic polymers. **a** Structure of electromechanical photonic crystals using dielectric elastomer actuator. **b** Photonic organogel is electrically operated with Maxwell stress induced by a DC bias, which results in areal expansion of the photonic gel. **c** Digital images show various colors from red to blue. **d** The electromechanical photonic device can generate sound in the audible frequency regime. **e** Piano notes to be programmed as an input signal to the device and recorded sound waves from a microphone. **f** A short-time Fourier transformation data, allowing for visualization of the frequencies over time. **g** Schematic structures for electro-mechano-chemically responsive(EMCR) color change displays. **h** Mechanism for the device using mechanochromic spiropyran materials. The applied voltages induce a large deformation in the elastomer, which causes ring open reaction of spiropyran resulting in color change (Figure reproduced from **a**–**f** [82], Copyright 2018, WILEY-VCH Verlag GmbH & Co. KGaA, Weinheim; **g**, **h** [83], Copyright 2014, Springer Nature)

Among electroactive polymers, dielectric elastomer actuators (DEA) [79–81] have attracted much attention due to their capacity to induce larger areal strains than existing electroactive polymers. This points to the possibility of integrating them with mechanochromic materials to achieve color changes. Kim et al. presented soft photonic devices fabricated by integrating DEA with 3D photonic organogel, which displays red, green and blue colors, by controlling the electric field (Fig. 11a). When voltages are applied to DEA, Maxwell stress occurs first, on the dielectric layer, and physical deformation due to the stress is conveyed to the contacting photonic gel, leading to a change in lattice distance (Fig. 11b). Photonic colors are reversibly modulated by tuning the DC voltages (Fig. 11c). They also demonstrate sound generation from the photonic device by exploiting the features of the color change mechanism based on the eletromechanical force, which enables the device to generate acoustic vibrations at higher frequencies (Fig. 11d). Sound wave composed of piano notes was used as an input signal for demonstration. Then, output sound from the device was recorded using a microphone and analyzed with a short-time Fourier transformation (Fig. 11e, f) [82]. Another study on the use of mechanochromic materials on DEA was reported by Qiming et al. They made an electro-mechano-chemically responsive (EMCR) elastomer containing spiropyran moieties. The elastomer layer is bonded to the buffer elastomer, which is in contact with an insulating layer to prevent electrical breakdown on the device (Fig. 11g). By applying voltages to the electrodes, large deformations occur on the elastomer layer, in a wrinkle pattern. This activates the ring opening reaction of spiropyran and causes the color to change (Fig. 11h) [83].

## Strategies for stretchable reflective display

### Polymeric substrates for stretchable reflective display

For stretchable displays, substrate technology is required to move beyond the conventional realm of rigid metals and ceramics. Hence, studies on polymer substrates are currently underway. Polymer substrates for displays must possess some optical and thermal properties, such as glass like transmittance (> 85%) and low constant of thermal expansion (CTE) [84, 85]. Most of all, it is important that the substrates have non-rigid mechanical properties, including elasticity.

**Fig. 12:**
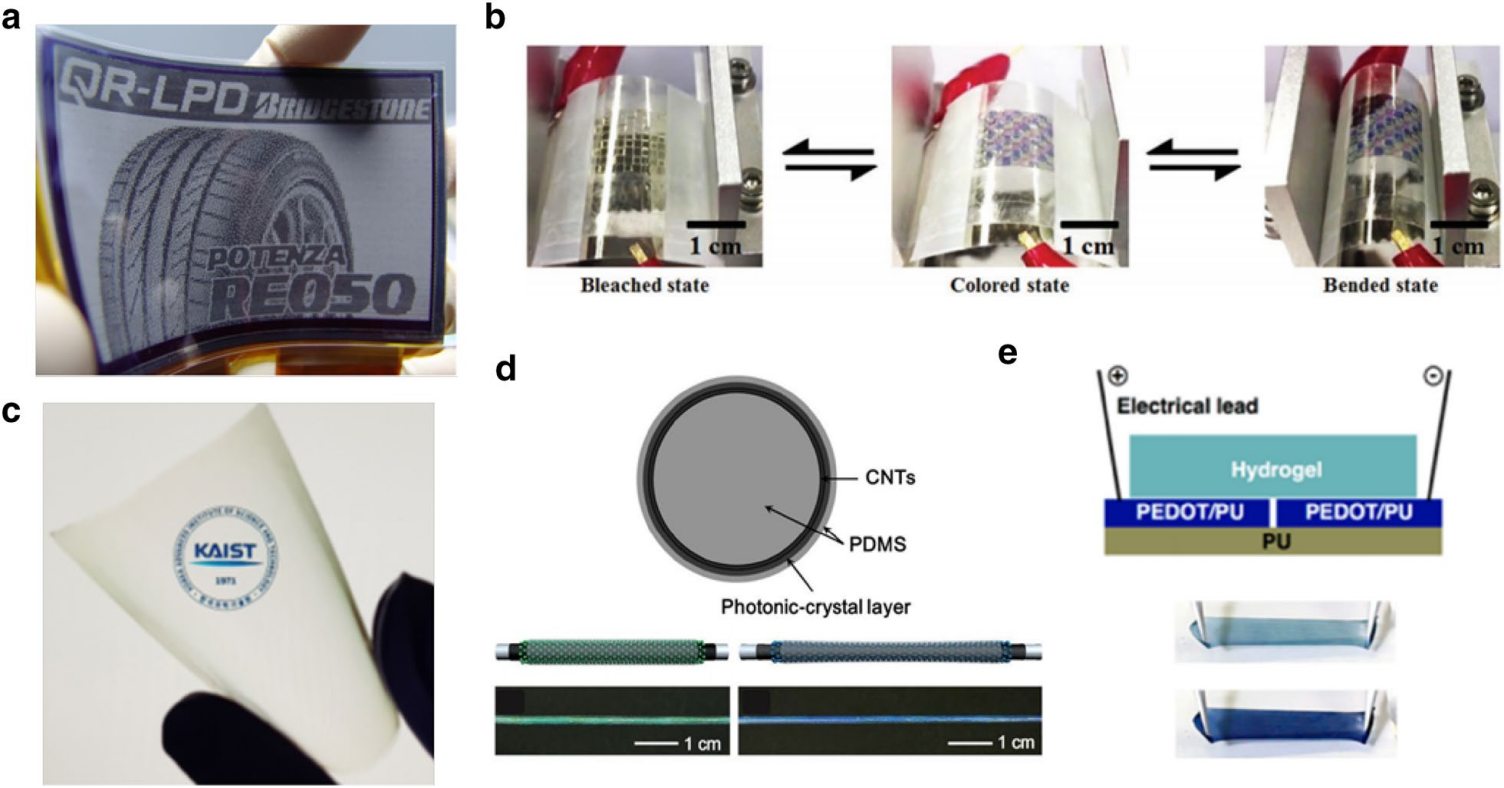
Polymeric substrates for stretchable displays. **a** Flexible E-paper display(QR-LPD^®^) fabricated on PET substrate. **b** Flexible electrochromic display on ITO coated PET substrate during dynamic bending test. **c** Picture of a poly amide-imide film with low CTE(~ 4 ppm/ °C) and high transparency. **d** Schematic structure of photonic crystal fiber and color change at given strain. Polystyrene particles are coated on PDMS core. **e** Stretchable electrochromic device fabricated on polyurethane(PU) substrate (Figure reproduced from **a** [18], Copyright 2006, Society for Information Display; **b** [35], Copyright 2019, WILEY-VCH Verlag GmbH & Co. KGaA, Weinheim; **c** [85], Copyright 2018, The Author(s); **d** [90], Copyright 2015, WILEY-VCH Verlag GmbH & Co. KGaA, Weinheim; **e** [91], Copyright 2017, American Chemical Society)

Soft elastomeric substrates can be a good option for stretchable displays. However, these materials are not easy to handle due to their low-dimensional stability, so a careful and delicate process is required during their fabrication or usage. Hence, prior to developing stretchable displays, flexible displays were studied using transparent and flexible polymers with relatively large dimensional stability in comparison to elastomers. PET is a typical polymer substrate for flexible displays due to its outstanding flexibility (can be bent over a 1-in diameter 1000 times), affordable price, high transparency (> 85%), and chemical resistance [84, 86, 87]. Many flexible displays use PET for substrates [18, 35] (Fig. 12a, b). However, PET exhibits relatively large CTEs in comparison to ceramics and metals because thermoplastic polymers have weak crosslinkings between chains [85]. Hence, obtaining lower CTEs for improved thermal stability has been an issue. For example, poly amide-imide thermoplastic film with a lower CTE (~ 4 ppm/ °C) is obtained by changing the ratio of two isomeric monomers without losing the high transmittance [85] (Fig. 12c).

As stretchable displays have started to attract the attention of the industrial world due to their potential applicability to human–machine interfaces, researchers have tried to apply established display technologies to stretchable materials. However, polymeric substrates for flexible displays have excessive Young’s moduli precluding applications to stretchable displays (GPa scale; typically 5 GPa for PET). New polymers are now needed for stretchable displays, and several transparent elastomers have been proposed as strong candidate substitutes for conventional thermoplastic substrates. PDMS has many favorable properties, such as biocompatibility, transparency, high electrical resistivity (~ 2.9 × 10^14^ Ω cm) and low Young’s modulus (~ 1 MPa) [88, 89]. It is therefore widely used in electronics, microfluidics and many other fields. Another candidate is polyurethane (PU), which has a low Young’s modulus (~ 5 MPa), large tear strength and abrasion resistance, which is required for devices that are frequently exposed to scratches or impact [89]. Both elastomers have been used as substrates for stretchable displays [90, 91] (Fig. 12d, e). However, there are still challenges to be addressed before these attractive polymers can be used as substrates for stretchable displays. For example, PDMS and PU exhibit larger CTE values (typically about 480 ppm/ °C for PDMS, 90–100 ppm/ °C for PU) even compared to PET. More importantly, the best way to secure sufficient dimensional stability for actual devices, despite their low elastic modulus, is not well understood. Trials to solve such issues will be required before we can use stretchable displays in practical applications.

### Stretchable electrodes for electrochemical reaction driven stretchable display systems

**Fig. 13:**
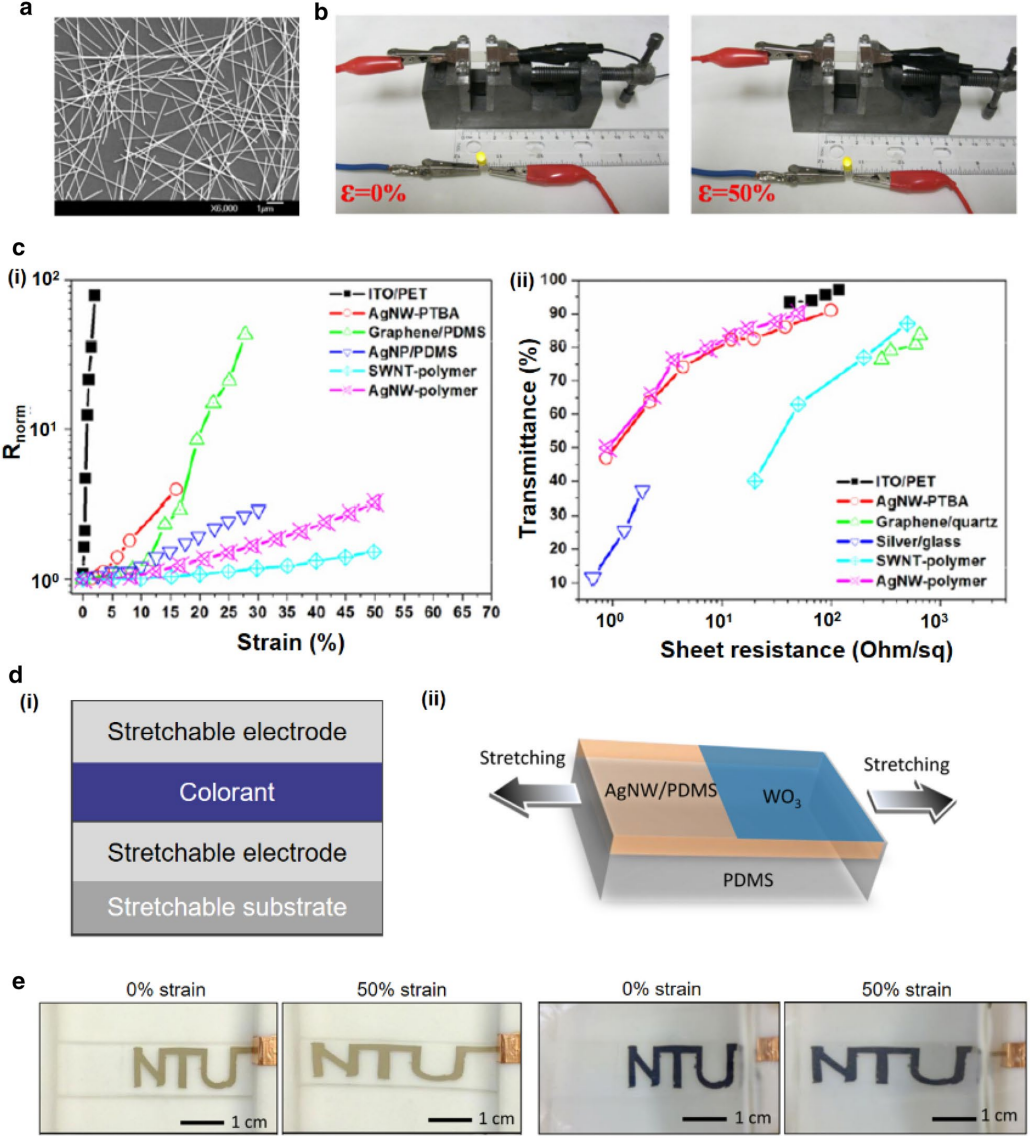
Stretchable electrodes and structure for stretchable electrochromic displays. **a** A SEM image of the AgNWs on a glass substrate. **b** Stretchability of a AgNW electrode is demonstrated with a light-emitting diode. **c** Electrical and optical properties of stretchable electrodes. (i) Normalized resistance as a function of applied strain. (ii) Transmittance versus sheet resistance. **d** (i) Possible structure of the stretchable electrochromic device. (ii) An example with AgNW/PDMS and WO_3_. **e** Patterned electrochromic device in bleached and colored states at 0 and 50% strain (Figure reproduced from **a**–**c** [92], Copyright 2012, IOP Publishing Ltd; **d**(ii) and **e** [94], Copyright 2013, America Chemical Society)

Electrochromic displays require electrodes that can exchange electrons, thus enabling electrochemical reactions. However, transparent electrodes, such as conventional ITO/PET complexes, are not suitable for stretchable displays because they can fail even under small strains. Thus, there have been some studies on how to fabricate material that is transparent and has sufficient electrical conductivity. Such properties have been achieved using silver nanowires (AgNWs) (Fig. 13a) embedded in the surface layer of polymers. Stretchable, transparent composites were synthesized with an AgNW network and crosslinked poly(acrylate) matrix [92]. This composite has a surface conductance and transparency comparable to that of ITO. The sheet resistance of the composite increased 2.3 times at 50% strain compared to its normal state (Fig. 13b) [92]. Furthermore, composites with conductive filler materials like carbon nanotubes (CNTs) [93], elastic conductors and metal nanoparticles can be exploited for stretchable electrodes with tensile strength over 100%. However, these materials have a rather high sheet resistance, of 100–1000 Ohm/sq at 80% optical transmittance. The sheet resistance, transmittance, and stretchability of the composite electrodes was compared to various conducting materials such as AgNWs, silver coating, single-wall carbon nanotubes (SWNTs), graphene, and ITO. The normalized resistance increased with the applied strain due to geometric changes during stretching (Fig. 13di). The transmittance of the material decreases with the sheet resistance (Fig. 13dii) [92].

Transparency and conductivity are both important for the top electrode of the stretchable display. Thus, it is possible to construct a structure with electrochromic materials sandwiched between two electrodes with sufficient transparency, conductivity, and mechanical properties (Fig. 13d). There have been some cases in which electrochromic molecules were combined with stretchable substrates [94, 95]. Stretchable electrochromic devices based on an AgNW/PDMS elastic conductor have been reported (Fig. 13dii) with an electrochemically deposited WO_3_ active layer. Fast coloration (1 s) and bleaching (4 s) times were achieved, and functioning at a 50% stretched state was demonstrated (Fig. 13e).

### Ionic conductors for electric field driven stretchable display systems

**Fig. 14:**
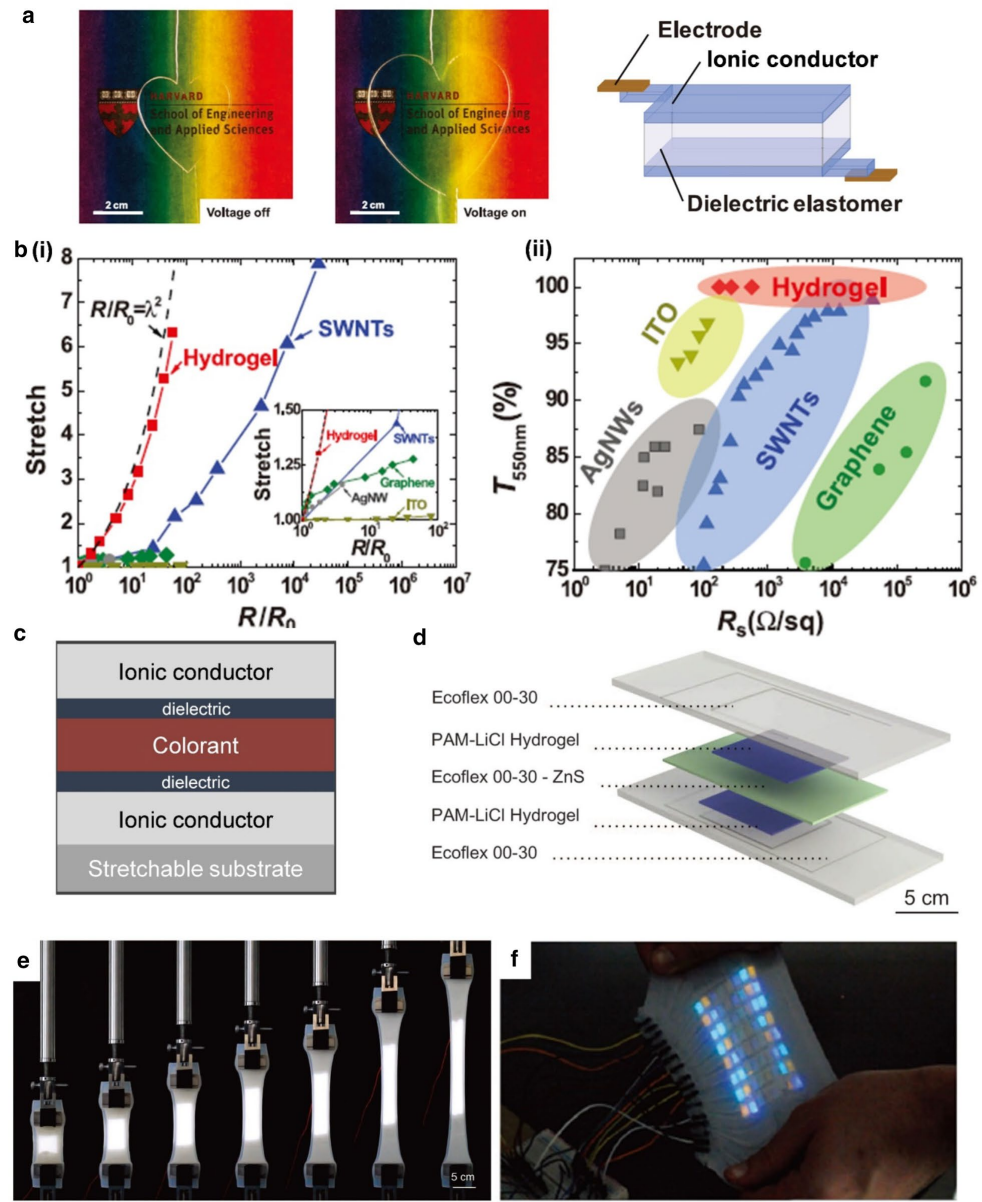
Ionic conductors for stretchable reflective displays. **a** Transparent ionic conductors are exploited for DEAs without electrochemical reaction. **b** Performance of hydrogel ionic conductor exhibiting relatively insensitive resistance change upon stretching with a high transparency comparing to other electrodes. **c** A design for stretchable reflective display with ionic conductors. **d** A similar structure of electroluminescent display using ionic conductors. **e** Luminescent behavior of the display under uniaxial stretching. **f** Patterned luminescent displays showing various colors under a mechanical deformation (Figure reproduced from **a**, **b** [96], Copyright 2013, American Association for the Advancement of Science; **d**, **e** [96], Copyright 2016, American Association for the Advancement of Science; f [97], Copyright 2016, WILEY-VCH Verlag GmbH & Co. KGaA, Weinheim)

There is the other system driving stretchable reflective displays that control and display colors by an electric field. Electric field-, rather than electron transport-driven systems have dielectric layers separating two electrodes, like a capacitor. In this case, in which only electric fields are exploited, ionic conductors can replace electrodes, which allows the system to take advantage of ionic conductors. Sun et al. demonstrated the operation of an electroactive device without electrochemical reactions by taking advantage of ionic conductors [96]. Electrochemical reactions are a major concern when ions are exploited in conjunction with applied voltages. The device with capacitor structures is transparent in the visible light range and exhibits electrical actuation with areal stretching when voltages are applied (Fig. 14a).

Hydrogel ionic conductors have lower sheet resistance and maintain high transmittance with relatively insensitive resistance changes under stretching in comparison to other electrodes, such as AgNWs, SWNTs, ITO and graphene (Fig. 14b). Also, ionic conductors are highly stretchable, easy to make and inexpensive, which is important for the fabrication of commercial stretchable displays. Thus, a design for stretchable reflective displays using ionic conductors has been proposed (Fig. 14c). By introducing colorants such as pigments or mechanochromic materials between the transparent dielectric elastomer, a colored dielectric layer can be fabricated. Subsequently, the layer is sandwiched between two transparent ionic conductors with a stretchable substrate. Even though the design seems feasible for stretchable reflective displays, no demonstrations have yet been reported during the current development phase of reflective displays, which has proven slower than the development phase of emissive displays. A similar design using electroluminescent materials was presented for stretchable emissive displays by Larson et al. (Fig. 14d) [97]. The display consists of an electroluminescent dielectric layer that is fabricated by mixing zinc sulfide phosphor into EcoFlex. The dielectric layer is then sandwiched between two ionic conductors and operated by applying a voltage, and emits white light continuously even under uniaxial stretching to over 395% strain (Fig. 14e). Also, by using a patterning technique, stretchable multicolor displays have been demonstrated under mechanical deformation (Fig. 14f) [98]. In the same manner, reflective materials that are used in electrophoretic, electrokinetic and electromechanical color change systems can be used in conjunction with ionic conductors for stretchable reflective displays.

### Issues in fabrication of Stretchable reflective display

**Fig. 15:**
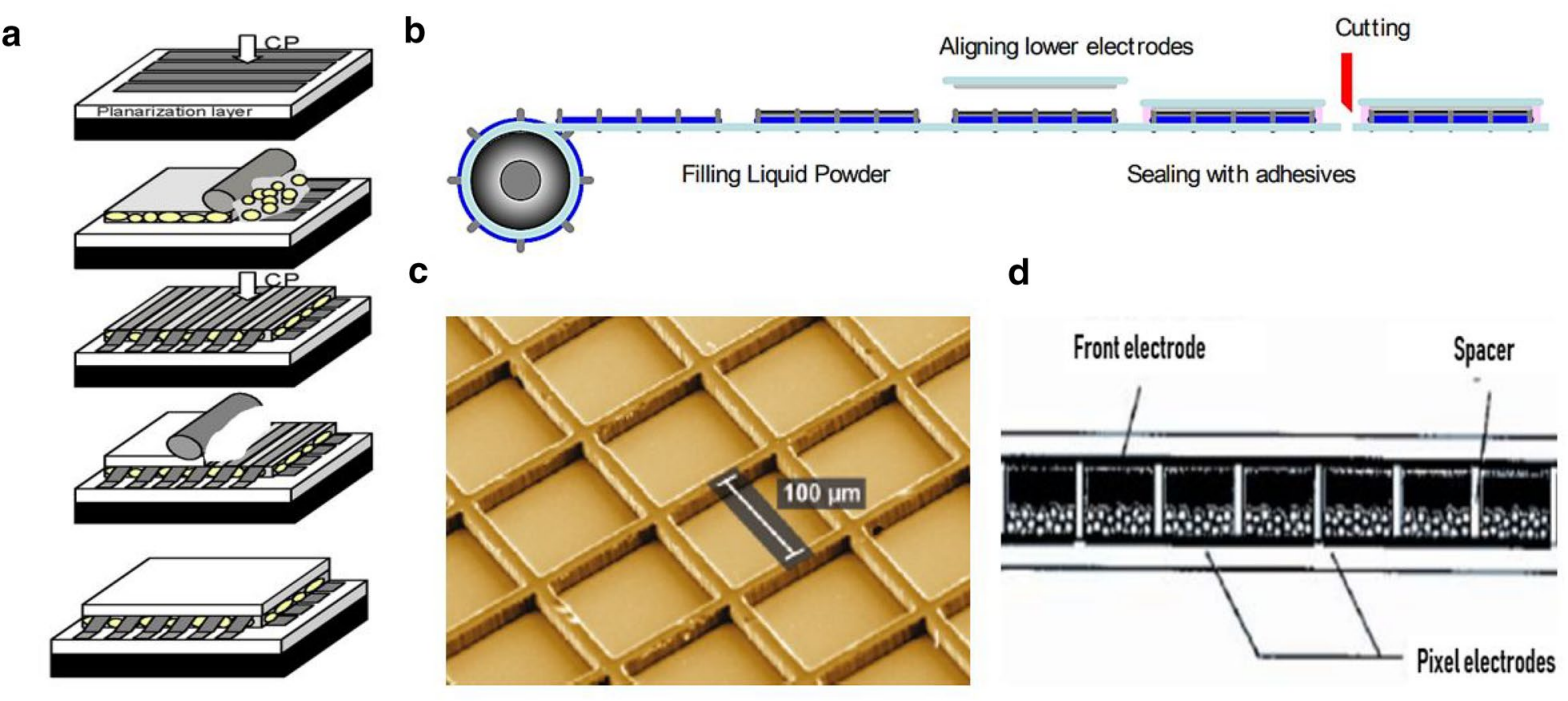
Display fabrication processes and pixel structures. **a** Schematic process of layer-to-layer fabrication of the reflective display, reproduced with permission. **b** Roll-to-roll process for liquid powder display fabrication. **c** ITO grid patterned by lithography. **d** Structure of electrophoretic display with spacers (Figure reproduced from **a** [99], Copyright 2006, Society for Information Display; **b** [18], Copyright 2006, Society for Information Display; **c** [101], Copyright 2010, American Chemical Society; **d** [101], Copyright 2005, John Wiley & Sons, Ltd)

All stretchable displays are basically composites of soft materials. Being similar to other stretchable emissive displays, stretchable reflective displays are composed of top/bottom substrates, two electrodes and a display working layer. Such components are arranged vertically in most displays, so layer-to-layer processes are typical fabrication methods [99] (Fig. 15a). Bottom substrates are prepared first, followed by a planarization process if required. Then, the lower electrode and display layer, upper electrode and top substrate are laminated one by one. Roll-to-roll manufacturing processes have been used to product displays more efficiently. This process has high throughput, and has already been adopted for flexible reflective displays [18] (Fig. 15b). In both processes mentioned, separating each pixel is important. Photolithography can be used to pattern the grid for each pixel [100] (Fig. 15c). Also there are spacers in the display layer, to separate each pixel. These spacers are connected to both upper/lower patterned electrodes, completely closing each pixel [101] (Fig. 15d). Here, the problem is that such connections between different materials (i.e., between substrates, electrodes, display layers or spacers) can be vulnerable to mechanical failure or invasion by impurities when the display is stretched.

**Fig. 16:**
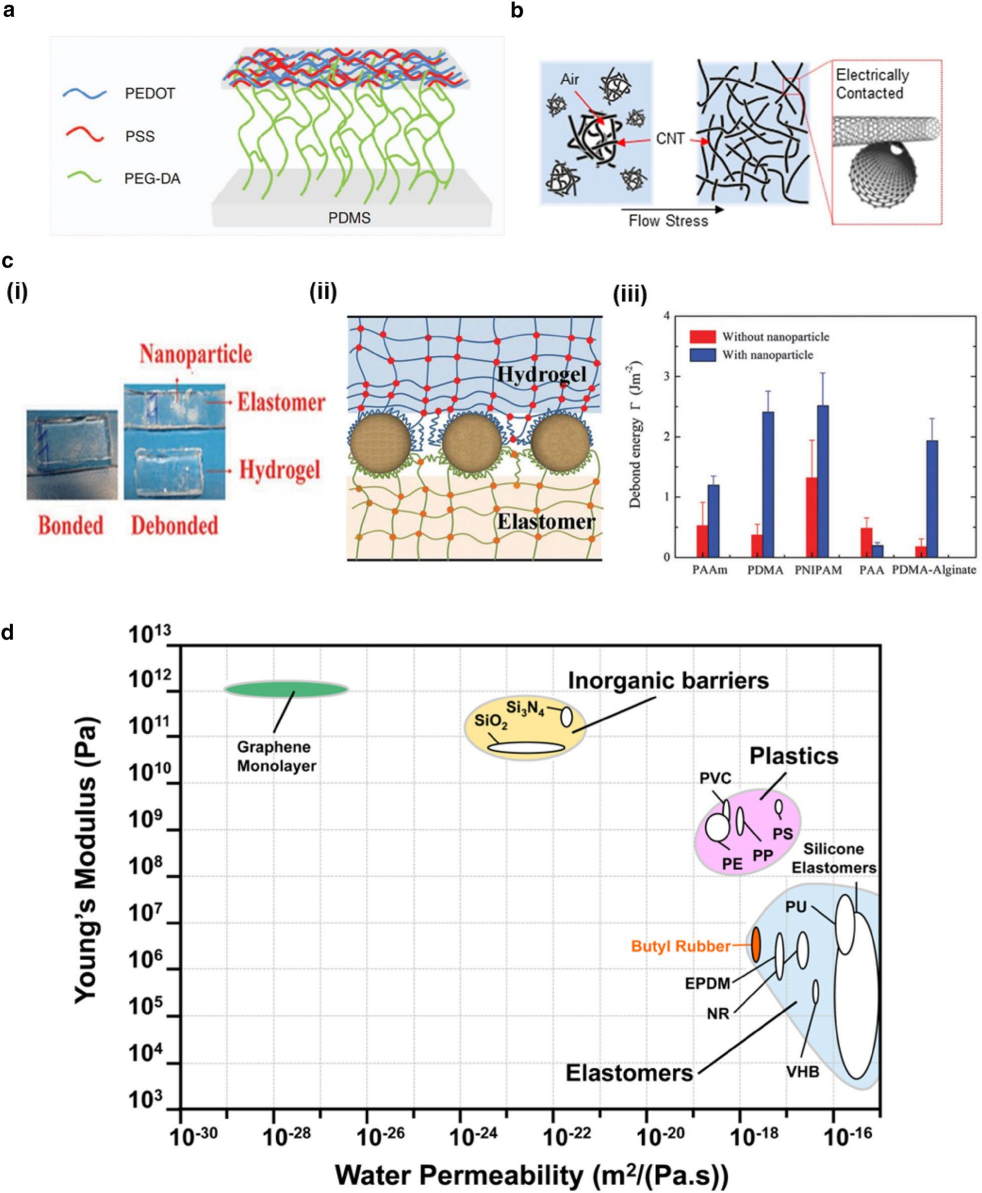
Adhesion and sealing issues. **a** PEDOT: PSS and PDMS is chemically linked by poly ethyleneglycol diacrylate (PEGDA). **b** CNT dispersion into PDMS by flow stress. **c** (i) Bilayer of VHB 4910 (3 M) elastomer and hydrogel with silica nanoparticles, before and after debond. (ii) Nanoparticles absorb chains between hydrogel and elastomer. (iii) Debond energies between VHB elastomer and various hydrogels are increased by silica nanoparticles. **d** water permeability and elastic modulus of variety of materials (Figure reproduced from **a** [102], Copyright 2017, The Author(s); **b** [103], Copyright 2014, The Author; **c** [104], Copyright 2016, The Royal Society of Chemistry; **d** [105], Copyright 2017, American Chemical Society)

When stretched simultaneously, materials with different moduli cause non-uniform stress fields. Hence, when different materials are adhered to make a stretchable display, tight adhesion is necessary to prevent mechanical failure. In stretchable displays, most structural materials are elastomers, so adhesion between elastomers and other materials is of paramount importance. The most common way to adhere two different polymers is to use chemical linkages. In Fig. 16a, conductive PEDOT:PSS and PDMS are chemically linked by a poly (ethylene glycol) diacrylate (PEGDA) layer [102]. Such chemical linkages can be applied to many other polymer/polymer junctions. Simply mixing two materials is another common method for adhesion. Many stretchable electrodes are produced in this way, dispersing nanomaterials like CNTs or AgNWs into non-cured elastomers. Nanomaterials tend to aggregate with each other due to the van der Waals force. In Fig. 16b, aggregated CNTs are equally dispersed and embedded into PDMS by flow stress, forming electrical networks between CNTs [103]. Another type of adhesion is between elastomers and hydrogels. Hydrogels with salts can act as ionic conductors, which are transparent and stretchable. In this case, nanoparticles can be used to absorb chains from both the elastomer and hydrogel, linking them mechanically. In Fig. 16c, adhesion between very high bond (VHB) acrylic elastomer and PAAm hydrogel is reinforced by the mechanical linkages between silica nanoparticles [104].

Tight adhesion at inner interfaces is important for mechanical stability. Similarly, tight sealing at the external surface of a device is also very important for chemical stability. To prohibit molecules from passing through, surface materials should have low permeability to water, oxygen and any other materials. For example, hydrogel can be coated with butyl rubber to prevent water from evaporating [105]. Such coatings can protect entire displays from impurities, because butyl rubber has much lower water and oxygen permeability than typical PDMS and many of other elastomers [106]. Elastomers with low elastic moduli and low permeability should be selected as external coating materials to prevent the infiltration of impurities [105], and reduce the risk of damage to the stretchable reflective display (Fig. 16d).

## Conclusion

As human–machine interfaces are becoming increasingly seamless, the demand for wearable displays is also increasing. Reflective displays are strong candidates for wearable displays due to outdoor readability, but being stretchable is still a problem for wearable displays. We have classified stretchable reflective displays into two groups: stretch-insensitive passive displays and stretch-sensitive active displays. Candidates for passive displays, such as electrophoretic, electrofluidic, and electrochromic displays, have already been demonstrated in a flexible form, so passive displays are considered to be closer to practical realization than active displays. Active displays are attracting much attention due to the interactivity between deformation by the user and the color of the display. Many mechanochromic active displays are being tested but are still at the experimental stage.

We have analyzed several strategies for fabricating stretchable reflective displays. The ideal stretchable display is one in which every component is substituted with stretchable material. Elastomers, ionic conductors and stretchable electrodes with nanomaterials are all reasonable options. It is true that there are many challenges remaining for stretchable reflective displays. But, there has certainly been incremental progress made towards the development of stretchable materials and reflective displays. Even though stretchable displays are not expected to materialize in the near future, they will be achieved at some point. Given this potential, we implore researchers to continue to study stretchable reflective displays.

## Data Availability

The datasets used and/or analysed during the current study are available from the corresponding author on reasonable request.
